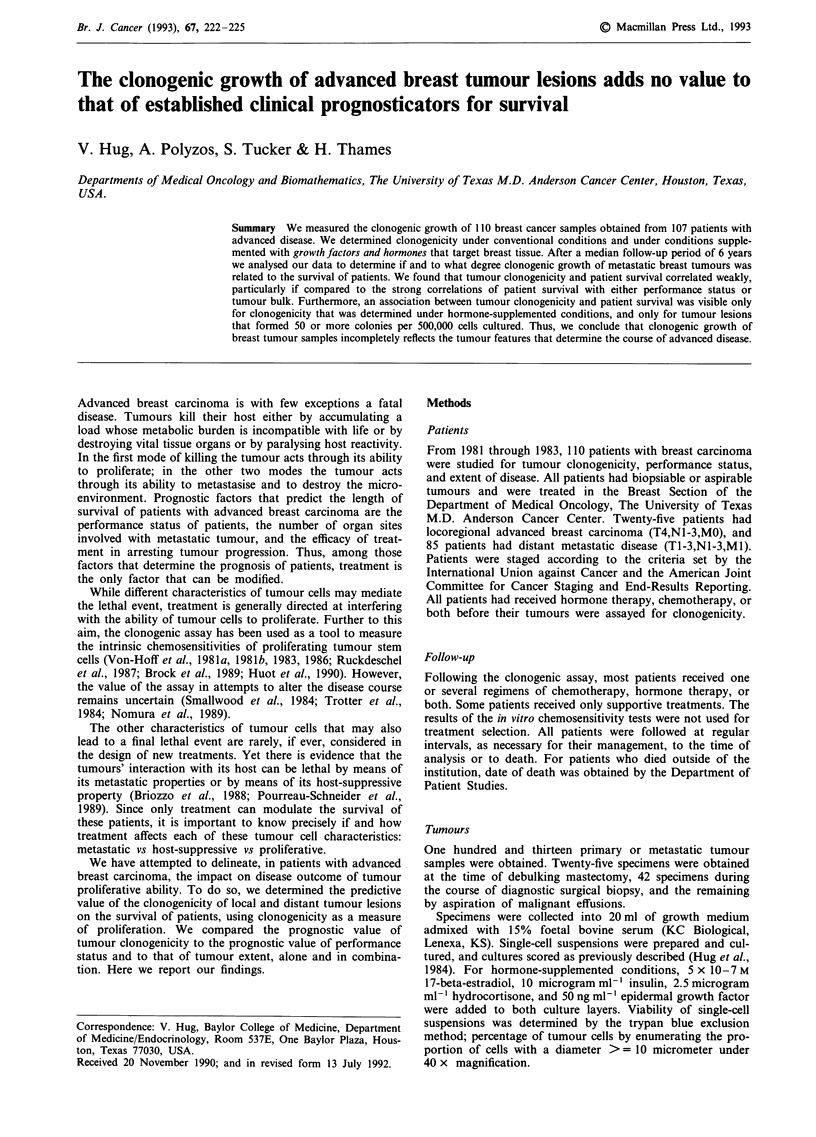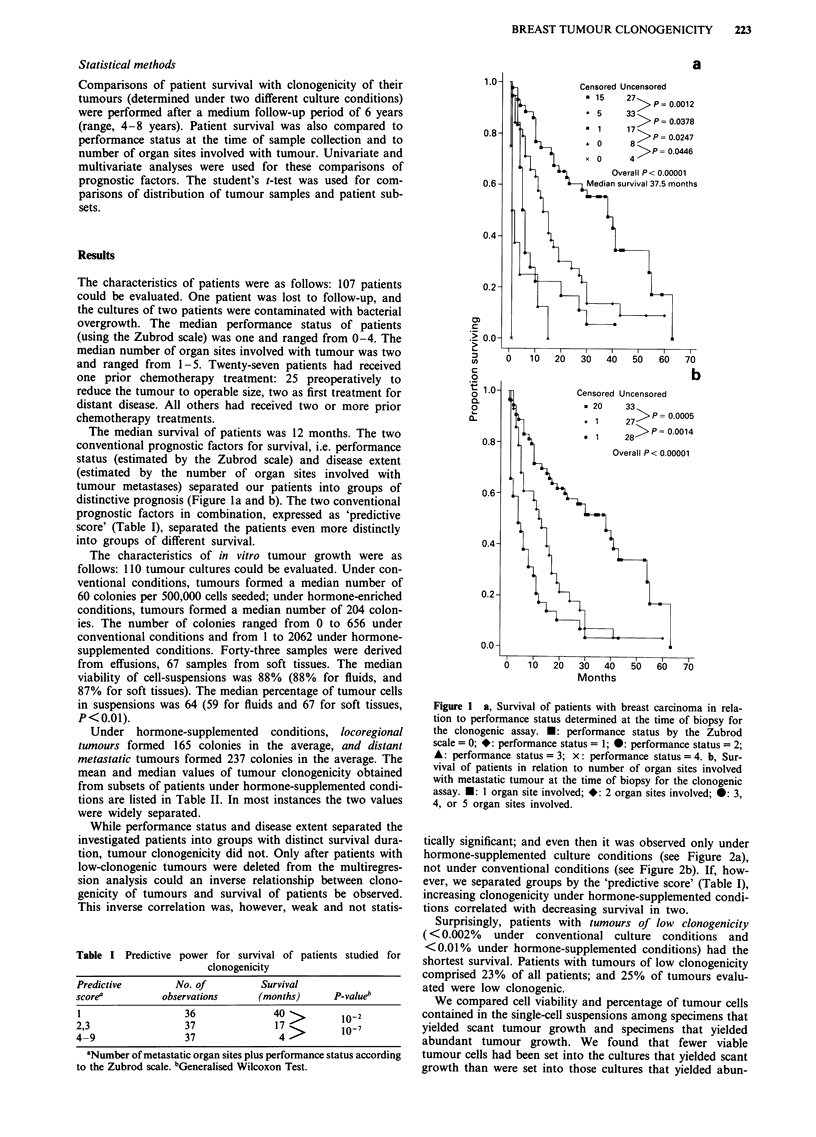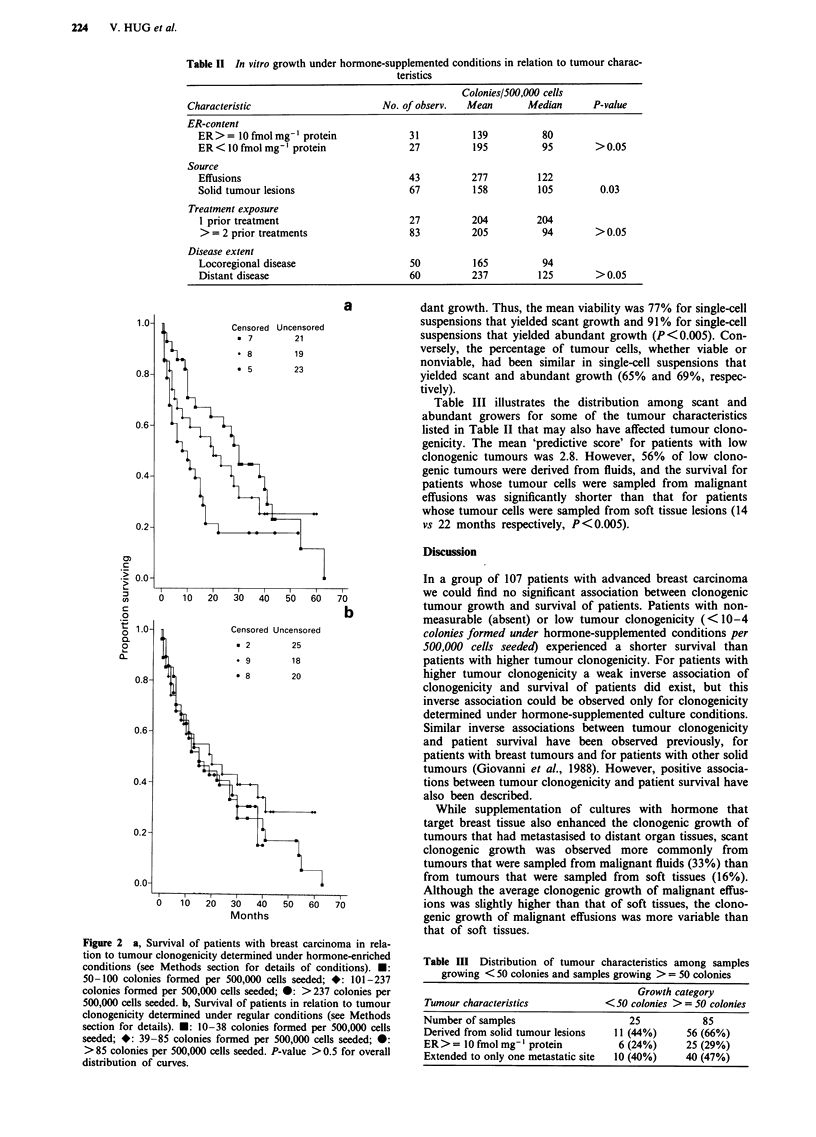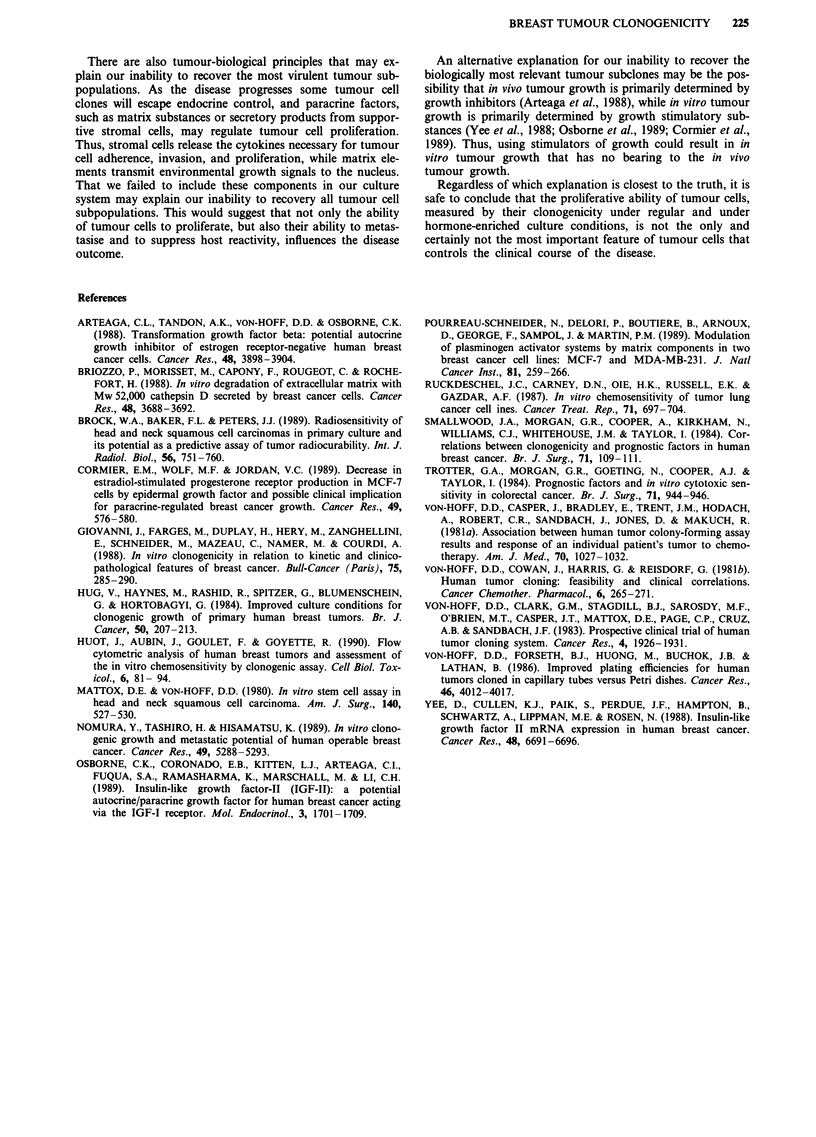# The clonogenic growth of advanced breast tumour lesions adds no value to that of established clinical prognosticators for survival.

**DOI:** 10.1038/bjc.1993.43

**Published:** 1993-02

**Authors:** V. Hug, A. Polyzos, S. Tucker, H. Thames

**Affiliations:** Department of Medical Oncology, University of Texas M.D. Anderson Cancer Center, Houston.

## Abstract

We measured the clonogenic growth of 110 breast cancer samples obtained from 107 patients with advanced disease. We determined clonogenicity under conventional conditions and under conditions supplemented with growth factors and hormones that target breast tissue. After a median follow-up period of 6 years we analyzed our data to determine if and to what degree clonogenic growth of metastatic breast tumours was related to the survival of patients. We found that tumour clonogenicity and patient survival correlated weakly, particularly if compared to the strong correlations of patient survival with either performance status or tumour bulk. Furthermore, an association between tumour clonogenicity and patient survival was visible only for clonogenicity that was determined under hormone-supplemented conditions, and only for tumour lesions that formed 50 or more colonies per 500,000 cells cultured. Thus, we conclude that clonogenic growth of breast tumour samples incompletely reflects the tumour features that determine the course of advanced disease.


Br. J. Cancer (1993), 67, 222–225 © Macmillan Press Ltd., 1993

### The clonogenic growth of advanced breast tumour lesions adds no value to that of established clinical prognosticators for survival

V. Hug, A. Polyzos, S. Tucker & H. Thames

*Departments of Medical Oncology and Biomathematics, The University of Texas M.D. Anderson Cancer Center, Houston, Texas, USA.*

**Summary** We measured the clonogenic growth of 110 breast cancer samples obtained from 107 patients with advanced disease. We determined clonogenicity under conventional conditions and under conditions supplemented with *growth factors and hormones* that target breast tissue. After a median follow-up period of 6 years we analysed our data to determine if and to what degree clonogenic growth of metastatic breast tumours was related to the survival of patients. We found that tumour clonogenicity and patient survival correlated weakly, particularly if compared to the strong correlations of patient survival with either performance status or tumour bulk. Furthermore, an association between tumour clonogenicity and patient survival was visible only for clonogenicity that was determined under hormone-supplemented conditions, and only for tumour lesions that formed 50 or more colonies per 500,000 cells cultured. Thus, we conclude that clonogenic growth of breast tumour samples incompletely reflects the tumour features that determine the course of advanced disease.

Advanced breast carcinoma is with few exceptions a fatal disease. Tumours kill their host either by accumulating a load whose metabolic burden is incompatible with life or by destroying vital tissue organs or by paralysing host reactivity. In the first mode of killing the tumour acts through its ability to proliferate; in the other two modes the tumour acts through its ability to metastasise and to destroy the microenvironment. Prognostic factors that predict the length of survival of patients with advanced breast carcinoma are the performance status of patients, the number of organ sites involved with metastatic tumour, and the efficacy of treatment in arresting tumour progression. Thus, among those factors that determine the prognosis of patients, treatment is the only factor that can be modified.

While different characteristics of tumour cells may mediate the lethal event, treatment is generally directed at interfering with the ability of tumour cells to proliferate. Further to this aim, the clonogenic assay has been used as a tool to measure the intrinsic chemosensitivities of proliferating tumour stem cells (Von-Hoff *et al.*, 1981*a*, 1981*b*, 1983, 1986; Ruckdeschel *et al.*, 1987; Brock *et al.*, 1989; Huot *et al.*, 1990). However, the value of the assay in attempts to alter the disease course remains uncertain (Smallwood *et al.*, 1984; Trotter *et al.*, 1984; Nomura *et al.*, 1989).

The other characteristics of tumour cells that may also lead to a final lethal event are rarely, if ever, considered in the design of new treatments. Yet there is evidence that the tumours' interaction with its host can be lethal by means of its metastatic properties or by means of its host-suppressive property (Briozzo *et al.*, 1988; Pourreau-Schneider *et al.*, 1989). Since only treatment can modulate the survival of these patients, it is important to know precisely if and how treatment affects each of these tumour cell characteristics: metastatic *vs* host-suppressive *vs* proliferative.

We have attempted to delineate, in patients with advanced breast carcinoma, the impact on disease outcome of tumour proliferative ability. To do so, we determined the predictive value of the clonogenicity of local and distant tumour lesions on the survival of patients, using clonogenicity as a measure of proliferation. We compared the prognostic value of tumour clonogenicity to the prognostic value of performance status and to that of tumour extent, alone and in combination. Here we report our findings.

Correspondence: V. Hug, Baylor College of Medicine, Department of Medicine/Endocrinology, Room 537E, One Baylor Plaza, Houston, Texas 77030, USA.
Received 20 November 1990; and in revised form 13 July 1992.

### Methods

### *Patients*

From 1981 through 1983, 110 patients with breast carcinoma were studied for tumour clonogenicity, performance status, and extent of disease. All patients had biopsiable or aspirable tumours and were treated in the Breast Section of the Department of Medical Oncology, The University of Texas M.D. Anderson Cancer Center. Twenty-five patients had locoregional advanced breast carcinoma (T4,N1-3,M0), and 85 patients had distant metastatic disease (T1-3,N1-3,M1). Patients were staged according to the criteria set by the International Union against Cancer and the American Joint Committee for Cancer Staging and End-Results Reporting. All patients had received hormone therapy, chemotherapy, or both before their tumours were assayed for clonogenicity.

### *Follow-up*

Following the clonogenic assay, most patients received one or several regimens of chemotherapy, hormone therapy, or both. Some patients received only supportive treatments. The results of the *in vitro* chemosensitivity tests were not used for treatment selection. All patients were followed at regular intervals, as necessary for their management, to the time of analysis or to death. For patients who died outside of the institution, date of death was obtained by the Department of Patient Studies.

### *Tumours*

One hundred and thirteen primary or metastatic tumour samples were obtained. Twenty-five specimens were obtained at the time of debulking mastectomy, 42 specimens during the course of diagnostic surgical biopsy, and the remaining by aspiration of malignant effusions.

Specimens were collected into 20 ml of growth medium admixed with 15% foetal bovine serum (KC Biological, Lenexa, KS). Single-cell suspensions were prepared and cultured, and cultures scored as previously described (Hug *et al.*, 1984). For hormone-supplemented conditions, 5 × 10–7 M 17-beta-estradiol, 10 microgram $ml^{-1}$ insulin, 2.5 microgram $ml^{-1}$ hydrocortisone, and 50 ng $ml^{-1}$ epidermal growth factor were added to both culture layers. Viability of single-cell suspensions was determined by the trypan blue exclusion method; percentage of tumour cells by enumerating the proportion of cells with a diameter >= 10 micrometer under 40 × magnification.

*Statistical methods*

Comparisons of patient survival with clonogenicity of their tumours (determined under two different culture conditions) were performed after a medium follow-up period of 6 years (range, 4–8 years). Patient survival was also compared to performance status at the time of sample collection and to number of organ sites involved with tumour. Univariate and multivariate analyses were used for these comparisons of prognostic factors. The student's *t*-test was used for comparisons of distribution of tumour samples and patient subsets.

### Results

The characteristics of patients were as follows: 107 patients could be evaluated. One patient was lost to follow-up, and the cultures of two patients were contaminated with bacterial overgrowth. The median performance status of patients (using the Zubrod scale) was one and ranged from 0–4. The median number of organ sites involved with tumour was two and ranged from 1–5. Twenty-seven patients had received one prior chemotherapy treatment: 25 preoperatively to reduce the tumour to operable size, two as first treatment for distant disease. All others had received two or more prior chemotherapy treatments.

The median survival of patients was 12 months. The two conventional prognostic factors for survival, i.e. performance status (estimated by the Zubrod scale) and disease extent (estimated by the number of organ sites involved with tumour metastases) separated our patients into groups of distinctive prognosis (Figure 1a and b). The two conventional prognostic factors in combination, expressed as 'predictive score' (Table I), separated the patients even more distinctly into groups of different survival.

The characteristics of *in vitro* tumour growth were as follows: 110 tumour cultures could be evaluated. Under conventional conditions, tumours formed a median number of 60 colonies per 500,000 cells seeded; under hormone-enriched conditions, tumours formed a median number of 204 colonies. The number of colonies ranged from 0 to 656 under conventional conditions and from 1 to 2062 under hormone-supplemented conditions. Forty-three samples were derived from effusions, 67 samples from soft tissues. The median viability of cell-suspensions was 88% (88% for fluids, and 87% for soft tissues). The median percentage of tumour cells in suspensions was 64 (59 for fluids and 67 for soft tissues, $P<0.01$).

Under hormone-supplemented conditions, *locoregional tumours* formed 165 colonies in the average, *and distant metastatic* tumours formed 237 colonies in the average. The mean and median values of tumour clonogenicity obtained from subsets of patients under hormone-supplemented conditions are listed in Table II. In most instances the two values were widely separated.

While performance status and disease extent separated the investigated patients into groups with distinct survival duration, tumour clonogenicity did not. Only after patients with low-clonogenic tumours were deleted from the multiregression analysis could an inverse relationship between clonogenicity of tumours and survival of patients be observed. This inverse correlation was, however, weak and not statistically significant; and even then it was observed only under hormone-supplemented culture conditions (see Figure 2a), not under conventional conditions (see Figure 2b). If, however, we separated groups by the 'predictive score' (Table I), increasing clonogenicity under hormone-supplemented conditions correlated with decreasing survival in two.

**Table I** Predictive power for survival of patients studied for clonogenicity

| *Predictive score*[a] | *No. of observations* | *Survival (months)* | *P-value*[b] |
|---|---|---|---|
| 1 | 36 | 40 | |
| | | | $10^{-2}$ |
| 2,3 | 37 | 17 | |
| | | | $10^{-7}$ |
| 4–9 | 37 | 4 | |

[a]Number of metastatic organ sites plus performance status according to the Zubrod scale. [b]Generalised Wilcoxon Test.

**Figure 1** **a**, Survival of patients with breast carcinoma in relation to performance status determined at the time of biopsy for the clonogenic assay. ■: performance status by the Zubrod scale = 0; ◆: performance status = 1; ●: performance status = 2; ▲: performance status = 3; ×: performance status = 4. **b**, Survival of patients in relation to number of organ sites involved with metastatic tumour at the time of biopsy for the clonogenic assay. ■: 1 organ site involved; ◆: 2 organ sites involved; ●: 3, 4, or 5 organ sites involved.

Surprisingly, patients with *tumours of low clonogenicity* (<0.002% under conventional culture conditions and <0.01% under hormone-supplemented conditions) had the shortest survival. Patients with tumours of low clonogenicity comprised 23% of all patients; and 25% of tumours evaluated were low clonogenic.

We compared cell viability and percentage of tumour cells contained in the single-cell suspensions among specimens that yielded scant tumour growth and specimens that yielded abundant tumour growth. We found that fewer viable tumour cells had been set into the cultures that yielded scant growth than were set into those cultures that yielded abun-

**Table II** *In vitro* growth under hormone-supplemented conditions in relation to tumour characteristics

| *Characteristic* | *No. of observ.* | *Colonies/500,000 cells* Mean | Median | *P-value* |
|---|---|---|---|---|
| *ER-content* | | | | |
| ER>= 10 fmol mg$^{-1}$ protein | 31 | 139 | 80 | |
| ER<10 fmol mg$^{-1}$ protein | 27 | 195 | 95 | >0.05 |
| *Source* | | | | |
| Effusions | 43 | 277 | 122 | |
| Solid tumour lesions | 67 | 158 | 105 | 0.03 |
| *Treatment exposure* | | | | |
| 1 prior treatment | 27 | 204 | 204 | |
| >= 2 prior treatments | 83 | 205 | 94 | >0.05 |
| *Disease extent* | | | | |
| Locoregional disease | 50 | 165 | 94 | |
| Distant disease | 60 | 237 | 125 | >0.05 |

**Figure 2** **a**, Survival of patients with breast carcinoma in relation to tumour clonogenicity determined under hormone-enriched conditions (see Methods section for details of conditions). ■: 50–100 colonies formed per 500,000 cells seeded; ◆: 101–237 colonies formed per 500,000 cells seeded; ●: >237 colonies per 500,000 cells seeded. **b**, Survival of patients in relation to tumour clonogenicity determined under regular conditions (see Methods section for details). ■: 10–38 colonies formed per 500,000 cells seeded; ◆: 39–85 colonies formed per 500,000 cells seeded; ●: >85 colonies per 500,000 cells seeded. *P*-value >0.5 for overall distribution of curves.

dant growth. Thus, the mean viability was 77% for single-cell suspensions that yielded scant growth and 91% for single-cell suspensions that yielded abundant growth ($P<0.005$). Conversely, the percentage of tumour cells, whether viable or nonviable, had been similar in single-cell suspensions that yielded scant and abundant growth (65% and 69%, respectively).

Table III illustrates the distribution among scant and abundant growers for some of the tumour characteristics listed in Table II that may also have affected tumour clonogenicity. The mean 'predictive score' for patients with low clonogenic tumours was 2.8. However, 56% of low clonogenic tumours were derived from fluids, and the survival for patients whose tumour cells were sampled from malignant effusions was significantly shorter than that for patients whose tumour cells were sampled from soft tissue lesions (14 *vs* 22 months respectively, $P<0.005$).

### Discussion

In a group of 107 patients with advanced breast carcinoma we could find no significant association between clonogenic tumour growth and survival of patients. Patients with non-measurable (absent) or low tumour clonogenicity (<10–4 *colonies formed under* hormone-supplemented conditions *per 500,000 cells seeded*) experienced a shorter survival than patients with higher tumour clonogenicity. For patients with higher tumour clonogenicity a weak inverse association of clonogenicity and survival of patients did exist, but this inverse association could be observed only for clonogenicity determined under hormone-supplemented culture conditions. Similar inverse associations between tumour clonogenicity and patient survival have been observed previously, for patients with breast tumours and for patients with other solid tumours (Giovanni *et al.*, 1988). However, positive associations between tumour clonogenicity and patient survival have also been described.

While supplementation of cultures with hormone that target breast tissue also enhanced the clonogenic growth of tumours that had metastasised to distant organ tissues, scant clonogenic growth was observed more commonly from tumours that were sampled from malignant fluids (33%) than from tumours that were sampled from soft tissues (16%). Although the average clonogenic growth of malignant effusions was slightly higher than that of soft tissues, the clonogenic growth of malignant effusions was more variable than that of soft tissues.

**Table III** Distribution of tumour characteristics among samples growing <50 colonies and samples growing >= 50 colonies

| *Tumour characteristics* | *Growth category* <50 colonies | >= 50 colonies |
|---|---|---|
| Number of samples | 25 | 85 |
| Derived from solid tumour lesions | 11 (44%) | 56 (66%) |
| ER>= 10 fmol mg$^{-1}$ protein | 6 (24%) | 25 (29%) |
| Extended to only one metastatic site | 10 (40%) | 40 (47%) |

There are also tumour-biological principles that may explain our inability to recover the most virulent tumour subpopulations. As the disease progresses some tumour cell clones will escape endocrine control, and paracrine factors, such as matrix substances or secretory products from supportive stromal cells, may regulate tumour cell proliferation. Thus, stromal cells release the cytokines necessary for tumour cell adherence, invasion, and proliferation, while matrix elements transmit environmental growth signals to the nucleus. That we failed to include these components in our culture system may explain our inability to recovery all tumour cell subpopulations. This would suggest that not only the ability of tumour cells to proliferate, but also their ability to metastasise and to suppress host reactivity, influences the disease outcome.

An alternative explanation for our inability to recover the biologically most relevant tumour subclones may be the possibility that *in vivo* tumour growth is primarily determined by growth inhibitors (Arteaga *et al.*, 1988), while *in vitro* tumour growth is primarily determined by growth stimulatory substances (Yee *et al.*, 1988; Osborne *et al.*, 1989; Cormier *et al.*, 1989). Thus, using stimulators of growth could result in *in vitro* tumour growth that has no bearing to the *in vivo* tumour growth.

Regardless of which explanation is closest to the truth, it is safe to conclude that the proliferative ability of tumour cells, measured by their clonogenicity under regular and under hormone-enriched culture conditions, is not the only and certainly not the most important feature of tumour cells that controls the clinical course of the disease.

### References

ARTEAGA, C.L., TANDON, A.K., VON-HOFF, D.D. & OSBORNE, C.K. (1988). Transformation growth factor beta: potential autocrine growth inhibitor of estrogen receptor-negative human breast cancer cells. *Cancer Res.*, **48**, 3898–3904.

BRIOZZO, P., MORISSET, M., CAPONY, F., ROUGEOT, C. & ROCHEFORT, H. (1988). *In vitro* degradation of extracellular matrix with Mw 52,000 cathepsin D secreted by breast cancer cells. *Cancer Res.*, **48**, 3688–3692.

BROCK, W.A., BAKER, F.L. & PETERS, J.J. (1989). Radiosensitivity of head and neck squamous cell carcinomas in primary culture and its potential as a predictive assay of tumor radiocurability. *Int. J. Radiol. Biol.*, **56**, 751–760.

CORMIER, E.M., WOLF, M.F. & JORDAN, V.C. (1989). Decrease in estradiol-stimulated progesterone receptor production in MCF-7 cells by epidermal growth factor and possible clinical implication for paracrine-regulated breast cancer growth. *Cancer Res.*, **49**, 576–580.

GIOVANNI, J., FARGES, M., DUPLAY, H., HERY, M., ZANGHELLINI, E., SCHNEIDER, M., MAZEAU, C., NAMER, M. & COURDI, A. (1988). *In vitro* clonogenicity in relation to kinetic and clinicopathological features of breast cancer. *Bull-Cancer (Paris)*, **75**, 285–290.

HUG, V., HAYNES, M., RASHID, R., SPITZER, G., BLUMENSCHEIN, G. & HORTOBAGYI, G. (1984). Improved culture conditions for clonogenic growth of primary human breast tumors. *Br. J. Cancer*, **50**, 207–213.

HUOT, J., AUBIN, J., GOULET, F. & GOYETTE, R. (1990). Flow cytometric analysis of human breast tumors and assessment of the in vitro chemosensitivity by clonogenic assay. *Cell Biol. Toxicol.*, **6**, 81– 94.

MATTOX, D.E. & VON-HOFF, D.D. (1980). *In vitro* stem cell assay in head and neck squamous cell carcinoma. *Am. J. Surg.*, **140**, 527–530.

NOMURA, Y., TASHIRO, H. & HISAMATSU, K. (1989). *In vitro* clonogenic growth and metastatic potential of human operable breast cancer. *Cancer Res.*, **49**, 5288–5293.

OSBORNE, C.K., CORONADO, E.B., KITTEN, L.J., ARTEAGA, C.I., FUQUA, S.A., RAMASHARMA, K., MARSCHALL, M. & LI, C.H. (1989). Insulin-like growth factor-II (IGF-II): a potential autocrine/paracrine growth factor for human breast cancer acting via the IGF-I receptor. *Mol. Endocrinol.*, **3**, 1701–1709.

POURREAU-SCHNEIDER, N., DELORI, P., BOUTIERE, B., ARNOUX, D., GEORGE, F., SAMPOL, J. & MARTIN, P.M. (1989). Modulation of plasminogen activator systems by matrix components in two breast cancer cell lines: MCF-7 and MDA-MB-231. *J. Natl Cancer Inst.*, **81**, 259–266.

RUCKDESCHEL, J.C., CARNEY, D.N., OIE, H.K., RUSSELL, E.K. & GAZDAR, A.F. (1987). *In vitro* chemosensitivity of tumor lung cancer cell ines. *Cancer Treat. Rep.*, **71**, 697–704.

SMALLWOOD, J.A., MORGAN, G.R., COOPER, A., KIRKHAM, N., WILLIAMS, C.J., WHITEHOUSE, J.M. & TAYLOR, I. (1984). Correlations between clonogenicity and prognostic factors in human breast cancer. *Br. J. Surg.*, **71**, 109–111.

TROTTER, G.A., MORGAN, G.R., GOETING, N., COOPER, A.J. & TAYLOR, I. (1984). Prognostic factors and *in vitro* cytotoxic sensitivity in colorectal cancer. *Br. J. Surg.*, **71**, 944–946.

VON-HOFF, D.D., CASPER, J., BRADLEY, E., TRENT, J.M., HODACH, A., ROBERT, C.R., SANDBACH, J., JONES, D. & MAKUCH, R. (1981*a*). Association between human tumor colony-forming assay results and response of an individual patient's tumor to chemotherapy. *Am. J. Med.*, **70**, 1027–1032.

VON-HOFF, D.D., COWAN, J., HARRIS, G. & REISDORF, G. (1981*b*). Human tumor cloning: feasibility and clinical correlations. *Cancer Chemother. Pharmacol.*, **6**, 265–271.

VON-HOFF, D.D., CLARK, G.M., STAGDILL, B.J., SAROSDY, M.F., O'BRIEN, M.T., CASPER, J.T., MATTOX, D.E., PAGE, C.P., CRUZ, A.B. & SANDBACH, J.F. (1983). Prospective clinical trial of human tumor cloning system. *Cancer Res.*, **4**, 1926–1931.

VON-HOFF, D.D., FORSETH, B.J., HUONG, M., BUCHOK, J.B. & LATHAN, B. (1986). Improved plating efficiencies for human tumors cloned in capillary tubes versus Petri dishes. *Cancer Res.*, **46**, 4012–4017.

YEE, D., CULLEN, K.J., PAIK, S., PERDUE, J.F., HAMPTON, B., SCHWARTZ, A., LIPPMAN, M.E. & ROSEN, N. (1988). Insulin-like growth factor II mRNA expression in human breast cancer. *Cancer Res.*, **48**, 6691–6696.